# Conjoined-network rendered stiff and tough hydrogels from biogenic molecules

**DOI:** 10.1126/sciadv.aau3442

**Published:** 2019-02-01

**Authors:** Liju Xu, Chen Wang, Yang Cui, Ailing Li, Yan Qiao, Dong Qiu

**Affiliations:** 1 Beijing National Laboratory for Molecular Sciences, State Key Laboratory of Polymer Physics and Chemistry, CAS Research/Education Center for Excellence in Molecular Sciences, Institute of Chemistry, Chinese Academy of Sciences, Beijing 100190, China.; 2 University of Chinese Academy of Sciences, Beijing 100190, China.

## Abstract

Hydrogels from biological sources are expected as potential structural biomaterials, but most of them are either soft or fragile. Here, a new strategy was developed to construct hydrogels that were both stiff and tough via the formation of the conjoined-network, which was distinct from improving homogeneity or incorporating energy dissipation mechanisms (double-network) approaches. Conjoined-network hydrogels stand for a class of hydrogels consisting of two or more networks that are connected by sharing interconnection points to collaborate and featured as follows: (i) All the composed networks had a similar or equal energy dissipation mechanism, and (ii) these networks were intertwined to effectively distribute stress in the whole system. As a specific example, a biogenic conjoined-network hydrogel was prepared by electrostatically cross-linking the chitosan-gelatin composite with multivalent sodium phytate. The combination of high compressive modulus and toughness was realized at the same time in the chitosan-gelatin-phytate system. Moreover, these physical hydrogels exhibited extraordinary self-recovery and fatigue resistance ability. Our results provide a general strategy for the design of biocompatible stiff and tough conjoined-network hydrogels due to a variety of potential cross-linking mechanisms available (e.g., electrostatic attraction, host-guest interaction, and hydrogen bonding).

## Teaser

By constructing a conjoined-network, we tackled the challenge in developing stiff and tough hydrogels of biogenic molecules.

## INTRODUCTION

The design and construction of hydrogels for wide applications in biomedical fields (*1*, *2*), such as tissue engineering (*3*, *4*), drug delivery (*5*), wound dressing (*6*), and structural implants (*7*, *8*), have attracted intensive attention. Because of the inherent biocompatibility and biodegradability of biogenic molecules, hydrogels based on polysaccharides (*9*–*11*), proteins (*12*–*14*), peptides (*15*, *16*), and DNA (*17*) are paving the way toward versatile application scenario. Under such a circumstance, onion-like chitosan hydrogels with intermembrane spaces for cell introduction (*9*), biostable d-amino acid residue hydrogels for intratumoral chemotherapy (*18*), and tunable alginate microgels encapsulating single cells for therapeutic delivery (*19*) have been demonstrated. Among these, little attention has been paid to biomedical load-bearing materials from biogenic hydrogels, for example, ligament, cartilage, and cell culture scaffold, because they are normally soft and brittle. Effective strategies to improve their mechanical properties are urgently needed.

Generally speaking, improving homogeneity and incorporating energy dissipation mechanisms have been recognized as effective methodologies to enhance mechanical performances of hydrogels. The ideally homogeneous network (involving spatial, topological, connectivity, and motility homogeneities) can behave cooperatively to avoid stress concentration, thus increasing the whole mechanical strength to some extent (*20*, *21*). However, due to the lack of effective energy dissipation mechanisms, significant improvement in mechanical properties cannot be achieved. On the other hand, incorporating energy dissipation mechanisms has proved to be more prominent (*22*–*31*). A typical case is the well-known double-network (DN) hydrogels (*23*, *26*, *29*, *30*), which combine two networks with heterogeneous structure and complementary properties and are denoted to have excellent mechanical properties. The first rigid and brittle network of DN hydrogels can effectively dissipate energy by scission of bonds, while the second soft and ductile network can withstand large strain to maintain the integrity of the hydrogel. Currently, DN hydrogels have been more dominating, and some derivatives are designed to improve them further, for instance, establishing connections between the two networks to distribute stress (*30*). However, because of the inhomogeneity between these two networks, the energy dissipation is mainly borne by the first network; therefore, increasing stiffness often deteriorates the toughness and vice versa (*32*, *33*), i.e., improving stiffness and toughness simultaneously remains a challenge.

**Fig. 1:**
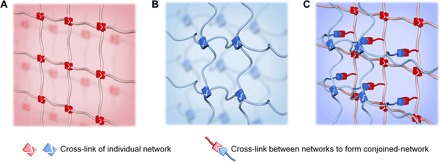
Structure features of a conjoined-network hydrogel. (**A** and **B**) A conjoined-network hydrogel composed of (A) the first network(s), which used to effectively consume energy by bond rupture, and (B) the second network(s) with similar energy dissipation mechanism to the first network(s) but lower cross-linking density, which involved dual functions: partaking energy dissipation with the first network(s) at the initial stage and maintaining the integrity of the hydrogel at the large deformation stage. (**C**) These networks were further intertwined with each other to form a conjoined-network, which effectively distributed stress in the whole system.

Different from the above two approaches, we designed conjoined-network hydrogels with synergistic energy dissipation mechanism to balance the inverse relation of stiffness and toughness (Fig. 1). Conjoined-network hydrogels stand for a class of hydrogels consisting of two or more networks that are connected by sharing interconnection points to collaborate and featured as follows: (i) All the composed networks had similar or equal energy dissipation mechanism, and (ii) these networks were intertwined to effectively distribute stress in the whole system. In detail, within conjoined-network hydrogels, at the initial deformation stage, bonds in all composed networks randomly broke to absorb energy, increasing the initial modulus significantly; at the larger deformation stage, one of these networks can withstand large strain attributed to the reduction of cross-linking density, promoting toughness effectively.

Here, we reported a particular example of conjoined-network hydrogels composed of biogenic molecules, including ionic cross-links of the first and second networks. In detail, a feasible “gelation and soaking” method was applied to convert weak chitosan-gelatin (C-G) composite hydrogels to stiff and tough chitosan-gelatin-phytate (C-G-P) conjoined-network hydrogels in sodium phytate solution. Sodium phytate, which has six phosphate groups, can interact with a dozen of amino groups of chitosan or gelatin chains to form two individual networks, and also couple these two networks by interacting with amino groups of both chitosan and gelatin simultaneously, resulting in a compact physical conjoined-network. The amine-phytate electrostatic domains played a dual role in enhancing the mechanical properties of hydrogels: reinforcing hydrogels by bond rupture and enhancing self-recovery and anti-fatigue capability by bond reformation. The gelation and soaking strategy was also a straightforward methodology to modulate the mechanical strength of hydrogels over broad ranges. Moreover, hydrogels were composed of biogenic molecules (chitosan, gelatin, and sodium phytate), which are well known to be biocompatible and biodegradable. A focus toward developing the conjoined-network should offer new directions for stiff and tough biomedical hydrogels based on other recoverable energy-dissipating mechanisms, such as host-guest interaction, hydrophobic interaction, and hydrogen bonding.

## RESULTS

### Construction of C-G-P physical conjoined-network hydrogels

**Fig. 2:**
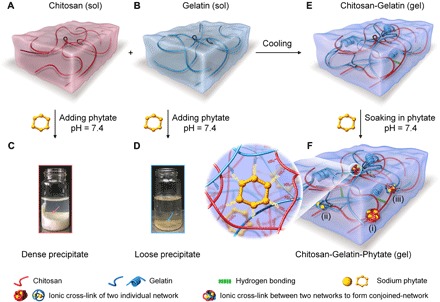
Construction of C-G-P physical conjoined-network hydrogel. (**A** and **B**) Scheme of (A) chitosan and (B) gelatin solution. (**C** and **D**) Optical photos of (C) the dense precipitate formed by chitosan [10 ml, 2 weight % (wt %)] with sodium phytate (1 ml, 20 wt %, pH 7.4) and (D) the loose precipitation formed by gelatin (10 ml, 20 wt %) with sodium phytate (1 ml, 20 wt %, pH 7.4). (**E**) C-G composite hydrogel. (**F**) C-G-P conjoined-network hydrogel. Domain (i) illustrates the first network consisting of the positively charged chitosan chains and phytate; domain (ii) reflects the second network built by the gelatin chains and phytate; and domain (iii) illustrates the preformed two networks that were further noncovalently linked with each other by phytate (photo credit: Liju Xu, Institute of Chemistry, Chinese Academy of Sciences).

Chitosan and gelatin (Fig. 2, A and B) were chosen as building blocks for biogenic conjoined-network hydrogels because they are inherently biocompatible and biodegradable and have abundant amino groups that can electrostatically interact with anions to form cross-linking points. As shown in Fig. 2C, direct addition of sodium phytate into chitosan solution led to dense precipitation, demonstrating the strong electrostatic interaction between chitosan (high density of amino groups) and phytate (high density of phosphate groups). Similarly, loose precipitation was obtained when gelatin solution was mixed with sodium phytate (Fig. 2D), indicating relatively weak electrostatic interaction between gelatin (low density of amino groups) and phytate.

To obtain hydrogels with phytate cross-linking, a gelation and soaking strategy was developed (*29*). The pre-prepared C-G composite hydrogel (Fig. 2E) by cooling mixed solution of chitosan and gelatin was used as a soft and brittle matrix and then soaked in sodium phytate solution (Fig. 2F). In this process, phytate ions permeated evenly and served as cross-linkers to form amino-phytate domains. This gelation and soaking protocol led to homogeneous hydrogels because the preformed gelatin network restricted the movement of the chitosan, thus preventing precipitation or coacervation. Scanning electron microscopy images confirmed the network formation in freeze-dried C4-G20-P0 and C4-G20-P40 hydrogels upon the sublimation of ice templates (fig. S1, A and B). It was revealed that after cross-linking, conjoined-network hydrogels became much denser than that of the C4-G20-P0 hydrogel. The resulting conjoined-network hydrogel with a 4.8-mm^2^ cross section can even bear a large load of 1 kg (fig. S1C). The conjoined-network plays a vital role in the remarkable comprehensive mechanical performances: (i) The high–amino density chitosan chains and phytate constituted the first network, effectively consuming energy by bond rupture to toughen the hydrogel; (ii) the low–amino density gelatin chains and phytate built up the second network, which involved dual mechanical functions: partaking energy dissipation at initial stage and maintaining the shape at large deformation stage; and (iii) those two networks were further noncovalently intertwined with each other by the co–cross-linking of phytate to effectively distribute stress in the whole system. Besides, the physical electrostatic amine-phytate domains introduced excellent self-recovery and anti-fatigue capability. To facilitate further discussion, we denoted C-G-P hydrogels using the abbreviation C*x*-G*y*-P*z*, where *x*, *y*, and *z* represented the original weight percentage of chitosan, gelatin in hydrogels, and sodium phytate in soaking solutions, respectively.

### Mechanical properties of C-G-P conjoined-network hydrogels

**Fig. 3:**
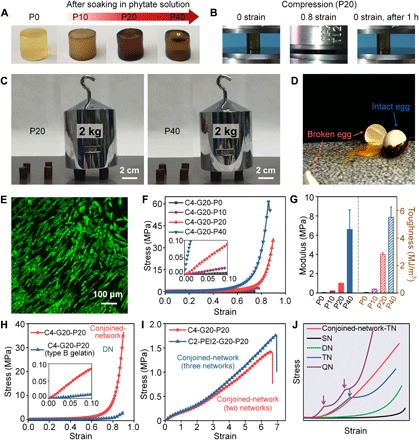
Mechanical properties of C-G-P conjoined-network hydrogels. (**A**) Optical images of C4-G20-P*z* hydrogels after soaking in sodium phytate solution with various concentrations (0, 10, 20, and 40 wt %; pH 7.4). (**B**) The C4-G20-P20 hydrogel can sustain a high compression strain of 0.8 and recover most of its original shape after relaxing for 1 hour. (**C**) C4-G20-P20 and C4-G20-P40 hydrogels can load a weight of 2 kg without any noticeable shape deformation. (**D**) The egg wrapped with the C4-G20-P20 hydrogel was dropped at 45 cm off the ground and kept intact, while the bare egg certainly broke into pieces. (**E**) Confocal fluorescence microscopy image of MC3T3-E1 cells cultured on the C4-G20-P20 hydrogel for 48 hours. (**F**) Compressive stress-strain curves (inset shows magnified plot in the low-strain region) and (**G**) modulus and compressive toughness of C4-G20-Pz hydrogels after soaking in sodium phytate solutions at various concentrations. The error bars represent SD; sample size *n* = 3. (**H**) Compressive performance of the C4-G20-P20 conjoined-network hydrogel and the C4-G20-P20 (type B gelatin) DN hydrogel. (**I**) Tensile property of the conjoined-network hydrogel composed of two and three networks. (**J**) Scheme of tensile stress-strain curve of the normal connected multiple-network hydrogel [single-network (SN), triple-network (TN), and quadruple-network (QN)] and the conjoined-network hydrogel incorporating three networks (conjoined-network triple network). Arrows show the strain localization in triple-network and quadruple-network hydrogels (photo credit: Liju Xu, Institute of Chemistry, Chinese Academy of Sciences).

The conjoined-network among chitosan, gelatin, and phytate endowed C-G-P hydrogels with outstanding mechanical properties suitable as structural materials. Homogeneous C4-G20-P*z* hydrogels (Fig. 3A) were obtained by soaking the preformed C4-G20 composite hydrogels in sodium phytate solution with various concentrations [10, 20, and 40 weight % (wt %)] at pH 7.4. As shown in Fig. 3B, the C4-G20-P20 hydrogel can be compressed to a strain of 0.8 without breaking and can recover to its original shape after removing the pressure within 1 hour. C4-G20-P20 and C4-G20-P40 hydrogels can maintain structural stability after loading a weight of 2 kg (approximately 500 times their own weight) without any noticeable deformation (Fig. 3C). This indicated that the hydrogel can be applied as a structural material to protect fragile objects, for example, an egg. As proof, an egg wrapped with the C4-G20-P20 hydrogel was dropped to the ground from a height of 45 cm and remained intact, while a bare egg from the same height broke into pieces (Fig. 3D and movies S1 and S2). In addition, both the preosteoblast MC3T3-E1 cells and human normal skin fibroblast cells cultured on the C4-G20-P20 hydrogel for 48 hours displayed almost entirely green fluorescence (Fig. 3E and fig. S2A). Quantitatively, the viability of human normal skin fibroblast cells cultured on the C4-G20-P20 hydrogel for 1, 4, and 7 days was determined to be 75 ± 8%, 80 ± 3%, and 91 ± 7%, respectively (fig. S2B), suggesting that the biogenic C4-G20-P20 hydrogel had good cytocompatibility in vitro. The C4-G20-P20 hydrogel can also be stretched over 200% strain and knotted with stretching over 300% strain (fig. S1, D and E). In contrast, the C4-G20-P0 hydrogel (C-G hydrogel without soaking) ruptured easily via compression or elongation (fig. S3, A and C) and cannot sustain a loading weight of 2 kg at all (fig. S3B).

The compressive properties of C-G-P conjoined-network hydrogels can be handily adjusted by varying soaking medium concentration. As shown in Fig. 3F, both the initial modulus and fracture strength enhanced with the increase of sodium phytate concentration, owing to the improvement of ionically cross-linking density of the conjoined-network. The compressive modulus of C4-G20-P20 and C4-G20-P40 hydrogels was significantly improved to 1.00 ± 0.04 MPa and 6.60 ± 2.04 MPa (Fig. 3G) compared to 0.06 ± 0.01 MPa of the C4-G20-P0 hydrogel. In addition, the compressive strengths of C4-G20-P20 and C4-G20-P40 hydrogels also reached 35.7 ± 1.0 MPa at a strain of 0.90 and 64.0 ± 6.4 MPa at a strain of 0.87, respectively, which were ~180 and ~320 times higher than that of the pristine C4-G20-P0 hydrogel (0.2 ± 0.1 MPa). Consequently, a marked increase in compressive toughness was also achieved, for example, from 0.04 ± 0.01 MJ/m^3^ for the C4-G20-P0 hydrogel to 2.80 ± 0.14 MJ/m^3^ and 5.50 ± 0.75 MJ/m^3^ for C4-G20-P20 and C4-G20-P40 hydrogels, respectively, accounting for an increase of 70 to 140 times (Fig. 3G). The tensile elastic modulus markedly increased from 0.03 ± 0.01 MPa for the composite C4-G20-P0 hydrogel to 0.46 ± 0.02 MPa and 2.47 ± 0.18 MPa for C4-G20-P20 and C4-G20-P40 hydrogels, respectively. In addition, tensile strengths of C4-G20-P20 and C4-G20-P40 hydrogels were 1.17 ± 0.08 MPa and 4.32 ± 0.94 MPa, respectively, which were 117 and 432 times higher than that of the C4-G20-P0 hydrogel (0.01 MPa) (fig. S1, F and G). In addition, the fracture energy (figs. S1H and S4) sharply increased to 5.31 ± 0.28 kJ/m^2^ and 13.79 ± 1.14 kJ/m^2^ for C4-G20-P20 and C4-G20-P40 hydrogels, respectively. The mechanical properties of all these biogenic hydrogels were much better than those of other biopolymer-based hydrogels (*10*, *11*, *34*–*36*) and comparable to those of synthetic tough DN hydrogels (*23*–*26*) or composite hydrogels (table S1) (*33*, *37*). They were also comparable to or even surpassed the mechanical properties of cartilage (*37*). Both high stiffness and toughness were realized at the same time in these hydrogels, attributed to the construction of the conjoined-network.

A control DN system consisting of chitosan-phytate type B gelatin was prepared to compare the properties of the DN hydrogel and the conjoined-network hydrogel. Unlike type A gelatin, type B gelatin (isoelectric point at 4.8 to 5.5) carries negative charges at pH 7.4, and therefore has weak interactions with the chitosan-phytate network. In Fig. 3H, the compressive strength of the C4-G20-P0 (type B gelatin) hydrogel before soaking is 0.13 ± 0.06 MPa, similar to that of the C4-G20-P0 (type A gelatin) hydrogel (0.20 ± 0.10 MPa). After soaking in phytate, the compressive strength of the C4-G20-P20 (type B gelatin) hydrogel slightly increased to 2.40 ± 0.70 MPa, much weaker than that of the C4-G20-P20 conjoined-network hydrogel (35.70 ± 1.00 MPa). This experiment proved that mechanical properties of the conjoined-network hydrogel can be higher than those of the DN hydrogel owing to the extra internetwork bonding.

In addition, the conjoined-network hydrogel incorporating multinetworks has a distinct shape in tensile stress-strain curve compared with the reported normal multinetwork hydrogels. As reported, normal chemically cross-linked triple- and quadruple-network hydrogels exhibit strain localization during deformation, which was an intriguing feature of the mechanical behavior as a consequence of local inhomogeneity of the cross-linking junctions (Fig. 3J) (*38*). However, the conjoined-network hydrogel incorporating three networks (C2-PEI2-G20-P20) was measured with high tensile strength and did not show this feature because composed networks were connected homogeneously and compactly (Fig. 3I).

The water contents of conjoined-network hydrogels were comparable to those of synthetic polymer hydrogels with similar toughness (*25*, *33*) or natural polymer hydrogels with much less toughness (*34*). With the increase of soaking medium concentration, the hydrogel water contents gradually decreased from 81 wt % for the C4-G20 composite hydrogel to 73, 60, and 51 wt % for C4-G20-P10, C4-G20-P20, and C4-G20-P40 conjoined-network hydrogels, respectively.

### Self-recovery and fatigue resistance of C4-G20-P20 conjoined-network hydrogel

**Fig. 4:**
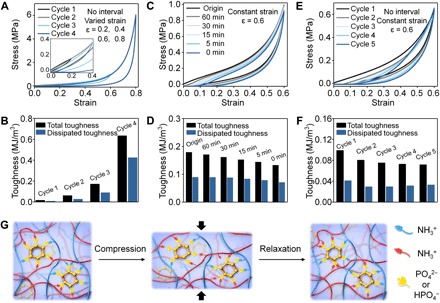
Self-recovery and fatigue resistance behavior of the C4-G20-P20 conjoined-network hydrogel. (**A**) Sequential loading-unloading compression tests without interval and (**B**) the corresponding calculated total and dissipated toughness of the C4-G20-P20 hydrogel under different strains (ɛ = 0.2, 0.4, 0.6, and 0.8). (**C**) Recovery cyclic compression tests and (**D**) the corresponding calculated total and dissipated toughness of the C4-G20-P20 hydrogel for different relaxation times under a constant strain (ɛ = 0.6). (**E**) Fatigue resistance and (**F**) the corresponding calculated total and dissipated toughness of the C4-G20-P20 hydrogel with five successive loading-unloading cycles without interval under a constant strain (ɛ = 0.6). (**G**) Proposed mechanism for the self-recovery and fatigue resistance ability: Ionic bonds reformed at original sites or other accessible sites.

As structural materials, besides sufficient modulus to maintain structural stability, self-recovery and fatigue resistance ability are also important for sustaining daily cyclic load and service life. Because of the reversible physically cross-linked networks, the C4-G20-P20 hydrogel had outstanding self-recovery and fatigue resistance. Upon cyclic loading of the C4-G20-P20 hydrogel with varied compression strain without interval between cycles, efficient energy dissipation was manifested as pronounced hysteresis loops (Fig. 4A). Generally, the area of hysteresis loops is used to measure the dissipated energy per unit volume, and the area becomes larger with increasing compression strain. At small strains (<0.4), the C4-G20-P20 hydrogel showed a tiny hysteresis loop. Larger area of hysteresis loop was found at strains of 0.6 and 0.8, indicating a large amount of energy being dissipated. Indeed, 0.09 MJ/m^3^ (51% of the total input work) and 0.42 MJ/m^3^ (67% of the total input work) were dissipated for the C4-G20-P20 hydrogel at strains of 0.6 and 0.8, respectively (Fig. 4B). In contrast, the C4-G20-P0 hydrogel showed much smaller hysteresis loops (fig. S5A), and only 0.003 MJ/m^3^ (29% of the total input work) was dissipated at a strain of 0.6 (fig. S5B). The large difference in hysteresis was ascribed to the effective energy dissipation provided by multiple ionic cross-linking among chitosan, gelatin, and phytate.

The recovery behavior of the C4-G20-P20 hydrogel after various relaxing times was summarized in Fig. 4 (C and D). We applied the area ratio of the second hysteresis loop to the first as the recovery efficiency and noticed that the recovery efficiency of the C4-G20-P20 hydrogel can exceed 98% after a relatively short relaxing time (~30 min). The anti-fatigue property of the C4-G20-P20 hydrogel was investigated by performing five cyclic loading-unloading compressive tests at a constant strain (0.6) without interval. There was no substantial plastic deformation and strength reduction. The dissipation energy slightly decreased from 0.051 for the first cycle to 0.037 for the second cycle (Fig. 4, E and F) and then remained virtually the same onward, i.e., 0.037, 0.039, and 0.041 MJ/m^3^ for the third, fourth, and fifth cycle, respectively. As a control, the C4-G20-P0 hydrogel exhibited substantial decreases in strength and dissipation energy in the cyclic tests (fig. S5, C and D). Unlike the gelatin hydrogel softened and even melted under human body temperature conditions (*34*), the C4-G20-P20 conjoined-network hydrogel showed good self-recovery (recovery efficiency can exceed 98% after 1 hour) and fatigue resistance ability (slight reduction of compressive strength and dissipated energy after five successive loading-unloading cyclic compression) at 37°C (fig. S6) compared to the behavior at 25°C. This property is expected to facilitate the applications of conjoined-network hydrogels in the field of biomedicine. These results suggest that the broken amino-phytate ionic cross-links can be healed after compression in a rather short time, at either the original sites or other accessible sites (Fig. 4G), which empowered the C4-G20-P20 hydrogel with excellent self-recovery and anti-fatigue capacity.

### Tunable mechanical properties of C-G-P conjoined-network hydrogels via adjustment of cross-linker functionality and weight ratio of the first network to the second network

**Fig. 5:**
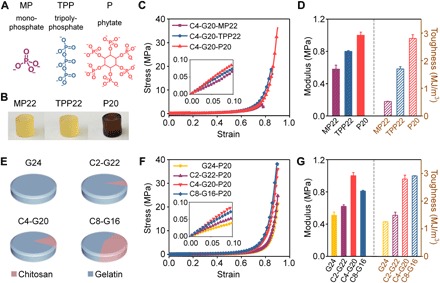
Tunable mechanical properties of C-G-P conjoined-network hydrogels via the adjustment of cross-linker functionality and weight ratio of the first network to the second network. (**A**) Chemical structures of phosphates with different phosphate group numbers. (**B**) Optical photos of C4-G20-MP22, C4-G20-TPP22, and C4-G20-P20 hydrogels. Photo credit: Liju Xu, Institute of Chemistry, Chinese Academy of Sciences. (**C**) Compressive stress-strain curves (inset shows magnified plot in the low-strain region) and (**D**) modulus and compressive toughness of C4-G20-MP22, C4-G20-TPP22, and C4-G20-P20 hydrogels. (**E**) Schematic of C*x*-G*y*-P20 hydrogels with different weight ratio of chitosan/gelatin after soaking in 20 wt % sodium phytate solution (pH 7.4). (**F**) Compressive stress-strain curves (inset shows magnified plot in the low-strain region) and (**G**) modulus and compressive toughness of C*x*-G*y*-P20 hydrogels with different weight ratio of chitosan/gelatin after soaking in 20 wt % sodium phytate solution (pH 7.4). The error bars represent SD; sample size *n* = 3.

Both functionality (number of cross-linkable sites in one cross-linker) and concentration of cross-linkers determine the cross-linking degree of the conjoined-network, thus affecting mechanical properties of hydrogels (*39*). In addition, we proposed that the functionality may play a more important role than concentration. To verify our hypothesis, we applied two other phosphates with a different number of phosphate groups as cross-linkers with the same total phosphate group concentration. As shown in Fig. 5A, monophosphate (MP) with one phosphate group potentially interacted with three amino groups, and tripolyphosphate (TPP) with three phosphate groups can interact with five amino groups, compared to phytate (P) with six phosphate groups (potentially interacted with 12 amino groups). After immersing in sodium dihydrogen phosphate and sodium tripolyphosphate solutions (molar concentrations of phosphate group identical to 20 wt % sodium phytate, pH 7.4), C4-G20-MP22 and C4-G20-TPP22 hydrogels also displayed enhancement in compressive modulus (0.58 ± 0.05 MPa for C4-G20-MP22 and 0.79 ± 0.01 MPa for C4-20-TP22), strength (4.1 ± 0.3 MPa for C4-G20-MP22 and 18.6 ± 0.3 MPa for C4-20-TP22), and toughness (0.52 ± 0.01 MJ/m^3^ for C4-G20-MP22 and 1.70 ± 0.08 MJ/m^3^ for C4-20-TP22), but they displayed lower enhancement compared to the C4-G20-P20 hydrogel (Fig. 5, B to D). Therefore, the enhanced mechanical property was highly dependent on the number of cross-linkable sites of one cross-linker. Namely, phytate with the most cross-linkable sites among these phosphates held the strongest electrostatic interaction to establish the most compact conjoined-network. This can be further confirmed by the greater amount of precipitate formed by direct mixing chitosan with phytate compared to mixing those with sodium dihydrogen phosphate or sodium tripolyphosphate (fig. S7, A and B).

Furthermore, the essential role of the ionically cross-linked conjoined-network in the macroscale mechanical properties of C-G-P hydrogels was also confirmed by adjusting the pH of sodium phytate. The change of sodium phytate solution pH from 7.4 to 3.5 or 12.2, which greatly weakened the electrostatic interaction between amino and phosphate, gave rise to the decrease in mechanical strength from 35.7 ± 1.0 MPa at pH 7.4 to 2.6 ± 0.1 MPa at pH 3.5 and 7.3 ± 1.1 MPa at pH 12.2 (fig. S7C). This is because at pH 3.5, most phosphate of phytate is neutral, and the electrostatic interaction was weakened; similarly, at pH 12.2, most amino was deprotonated and almost neutral.

Because of the different densities of amino group in chitosan and gelatin, the cross-linking degree of the conjoined-network can be adjusted by altering the weight ratio of chitosan to gelatin. At a fixed sodium phytate concentration of 20 wt %, the mechanical properties of C*x*-G*y*-P20 hydrogels were found to increase with chitosan ratio, i.e., increase of amino group density (Fig. 5, E to G), which was attributed to the increased phytate-amino cross-links. For instance, when the weight ratio of chitosan to gelatin increased from 0 to 0.2, the compressive modulus and toughness increased from 0.51 ± 0.04 MPa and 1.25 ± 0.01 MJ/m^3^ to 1.00 ± 0.04 MPa and 2.80 ± 0.14 MJ/m^3^, which further confirmed that the newly built conjoined-network can simultaneously increase stiffness and toughness. We also performed similar tests on those hydrogels at different weight ratios of chitosan and gelatin by adjusting the concentration of sodium phytate, and similar trends were observed (fig. S8, A to I). The conjoined-network hydrogels demonstrated moderate equilibrium swelling ratio in water ascribed to the compact structure. At a fixed sodium phytate concentration (20 wt %), the swelling ratio of C*x*-G*y*-P20 hydrogels was found to decrease with the chitosan ratio, i.e., increase of amino group density, which was attributed to the increased phytate-amino cross-links. For instance, when the weight ratio of chitosan to gelatin increased from 0 to 0.5, the swelling ratio decreased from 2.30 ± 0.12 to 1.10 ± 0.05, which further confirmed that the newly built conjoined-network can simultaneously decrease the swelling ratio (fig. S8, J and K).

It is worth mentioning that the pure gelatin hydrogel G80-P0 with approximately the same solid content to C*x*-G*y*-P20 hydrogels displayed much lower compression modulus (0.34 ± 0.01 MPa) and toughness (0.52 ± 0.07 MJ/m^3^) (fig. S9A), which further confirmed that the amino-phytate cross-links played an important role in the superb mechanical properties of C*x*-G*y*-P20 hydrogels. Meaningfully, the gelation and soaking protocol was facile and straightforward to tune the mechanical properties of hydrogels over a broad range, which can be readily achieved by adjusting the concentration of soaking media, the functionality of cross-linkers, and the weight ratio of the first network to the second network (fig. S9B).

### A universal approach to stiff and tough conjoined-network hydrogels

**Fig. 6:**
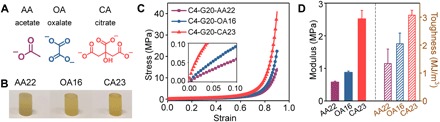
A universal approach to stiff and tough conjoined-network hydrogels. (**A**) Schematic structures of carboxylates with different numbers of carboxyl group. (**B**) Photographs of C4-G20-AA22, C4-G20-OA16, and C4-G20-CA23 hydrogels. Photo credit: Liju Xu, Institute of Chemistry, Chinese Academy of Sciences. (**C**) Compressive stress-strain curves (inset reveals magnified plot in the low-strain region) and (**D**) modulus and compressive toughness of C4-G20-AA22, C4-G20-OA16, and C4-G20-CA23 hydrogels. The error bars represent SD; sample size *n* = 3.

According to the reinforcing mechanism of C-G-P conjoined-network hydrogels, we proposed that immersing in anionic salt solutions is a universal method to convert the preformed C-G composite hydrogels into stiff and tough hydrogels via amino-anion cross-linking. To validate this hypothesis, we soaked C4-G20 composite hydrogels into a series of carboxylates with different numbers of carboxyl group (the molar concentration of carboxyl group was twice that of phosphate group at 20 wt % sodium phytate, pH 7.4; Fig. 6A). Similar to phosphate-soaked hydrogels, the obtained hydrogels (C4-G20-AA22, C4-G20-OA16, and C4-G20-CA23) showed enhancement in compressive modulus, strength, and toughness (Fig. 6, B to D). For example, the citrate-soaked C4-G20-CA23 hydrogel displayed the highest compressive modulus (2.52 ± 0.26 MPa), strength (39.2 ± 1.6 MPa), and toughness (3.07 ± 0.18 MJ/m^3^), owing to its highest number of carboxyl group. Therefore, the mechanical properties also increased with the number of carboxyl group per cross-linker, which was in good agreement with the previous conclusion drawn from the phosphate series. Imaginably, if we could find other suitable cross-linkers with multiple cross-linkable sites based on various mechanisms (e.g., electrostatic interaction, hydrogen bonding, and host-guest interaction), it is possible to obtain mechanically enhanced conjoined-network hydrogels for broader applications.

## DISCUSSION

To conclude, the conjoined-network hydrogel was constructed, which differed from the homogeneous network or the DN hydrogel in concept and topological structure: All the composed networks owned similar energy dissipation mechanism, and these networks were intertwined to effectively distribute stress in the whole system, which demonstrated a significant improvement in both modulus and toughness. As an example, a conjoined-network hydrogel composed of biogenic molecules using a gelation and soaking method was reported. The C-G-P hydrogels achieved an unusual combination of high modulus and toughness owing to the formation of the conjoined-network by chitosan, gelatin, and phytate. Moreover, the physical amino-phytate domains endowed the C-G-P hydrogels remarkable self-recovery and anti-fatigue capacity. It provided various facile strategies to tune mechanical properties over a wide range by adjusting the concentration of soaking media, the functionality of cross-linker, or the diverse ionic combination for various applications. In general, our results illustrate an approach to stiff and tough conjoined-network hydrogels with multifunction based on electrostatic interaction or other recoverable energy-dissipating mechanisms, such as hydrogen bonding and host-guest interaction. We believe that these conjoined-network hydrogels from biogenic sources can be applied in the fields of tissue engineering, vibration absorbers, soft robotics, and smart wearable devices.

## MATERIALS AND METHODS

### Materials

Chitosan (deacetylation degree ≥ 95%; viscosity, 100 to 200 mPa·s; Aladdin), sodium dihydrogen phosphate (Aladdin), sodium tripolyphosphate (Aladdin), gelatin (porcine skin, type A, 300 Bloom, Sigma-Aldrich), phytic acid (Sigma-Aldrich), and other chemicals (Sinopharm Chemical Reagent Co. Ltd.) were purchased and used without further purification.

### Preparation of C-G-P conjoined-network hydrogels

The gelation and soaking method was adopted to prepare C-G-P conjoined-network hydrogels. Briefly, different weights of chitosan (0.2, 0.4, and 0.8 g) and gelatin (2.2, 2.0, and 1.6 g) were dissolved in 10 ml of deionized water (1 wt % acetic acid) and stirred for 1 hour at 60°C. After removing air bubbles by vibrating on an oscillator, the transparent solutions were poured into Teflon molds and maintained at room temperature for 1 hour to form the original C-G composite hydrogels. Subsequently, the preformed hydrogels were soaked in sodium phytate solutions with the desired concentrations at pH 7.4 and at room temperature for 72 hours to obtain the C-G-P hydrogels.

### Mechanical test

All mechanical tests were performed with a universal tensile machine (Instron model 5567) in air, at room temperature, and at 30% humidity. The cylindrical hydrogels with a height of 10 mm and a diameter of 8 mm were used for compression tests at a speed of 1 mm/min. For the successive loading-unloading tests, the compression speed was set at 2 mm/min. For the successive loading-unloading tests under human body temperature condition, the test temperature was controlled at 37°C by air conditioner. The rectangular hydrogels (2 mm × 2 mm × 25 mm) were used for tensile tests at a speed of 50 mm/min. The strain of the hydrogel sample was estimated as the length change related to the initial length, and the stress was obtained by dividing the force by the initial cross-sectional area of the hydrogel sample. The elastic modulus was calculated using the slope of the initial linear region of the stress-strain curve. The toughness of hydrogels was evaluated by fracture energy and compressive toughness, wherein the fracture energy (J/m^2^) was measured by pure shear test and the compressive toughness (J/m^3^) was calculated as the area under stress-strain curves.

### Calculation of fracture energy

The rectangular hydrogels (75 mm × 1 mm × 5 mm) were used for the pure shear test to measure the fracture energy. Briefly, a force-displacement curve was measured for both the notched sample and unnotched sample with the same initial dimensions (the area of cross section of the sample is *A*) at the same test condition. The notched samples were prepared by cutting a notch with a length of 50% of the sample width by using a razor blade. *L*_c_ was defined as the critical distance where the notch turned into a running crack, and *U*(*L*_c_) is the work done to an unnotched sample to reach *L*_c_. The fracture energy is given by the following equation: Γ = *U*(*L*_c_)/*A*.

### Chitosan precipitate formation

Ten milliliters of 2 wt % chitosan solution was mixed with equal-volume phosphates, which have different phosphate group numbers (the molar concentrations of phosphate group were identical to those in 20 wt % sodium phytate, pH 7.4). The precipitate phase was allowed to settle down for 24 hours. The upper polymer-depleted equilibrium phase was removed from the dense precipitate phase by centrifugation. The precipitate phase was lyophilized and weighed. The content of chitosan in precipitate phase was obtained by thermogravimetric analysis (TA Instruments Pyris 1; from 25° to 650°C at a rate of 10°C/min under N_2_ environment). The chitosan coacervate ratio was calculated using Eq. 1

$$W_{\text{chitosan}}=\frac{m_{\text{chitosan precipitate}}}{m_{\text{total chitosan}}}\times 100\%$$ (1)

where *m*_chitosan precipitate_ is the weight of chitosan in the precipitate phase and *m*_total chitosan_ is the weight of chitosan in the original chitosan solution.

### Cell cultivation

The preosteoblast MC3T3-E1 cells (CRL-2593, American Type Culture Collection, Rockville, MD) and the human normal skin fibroblast cells (provided by Z. Qin from the Department of Plastic and Reconstructive Surgery at Peking University Third Hospital) were cultured on the surface of hydrogels at a density of 10^5^ cells/ml for 48 hours. LIVE/DEAD staining solution consisted of calcein-AM (2 μM), and propidium iodide (4 μM) solution was used to incubate hydrogels for 30 min at room temperature. Afterward, these hydrogels were washed three times with phosphate-buffered saline (PBS). Then, hydrogels were observed under a fluorescence microscope (Nikon C2-SIM, Japan). Cell viability was measured by the CCK-8 method. The human normal skin fibroblast cells were cultured in a 48-well plate with or without (control) the hydrogel with an initial density of 5 × 10^4^ cells/ml. At different intervals (1, 4, and 7 days), the culture medium was replaced with medium containing CCK-8 reagent and cells were subsequently incubated at 37°C for 3 hours. The optical density (OD) was measured at 450 nm [cell viability = OD_(with hydrogel)_/OD_(control)_].

### Scanning electron microscopy

The prepared hydrogels were frozen in liquid nitrogen and then freeze-dried at −50°C (FD-2C-80 Freeze dryer) to obtain aerogels. The resulting C4-G20-P40 hydrogel shrinked to about 70%. The freeze-dried aerogels were plunged into liquid nitrogen and ruptured, and the fresh cross sections were sputter-coated with a thin layer of platinum for scanning with a JSM-6700 scanning electron microscope (Japan) at 5 kV.

### Measurement of water content

The as-prepared hydrogels were first weighed (*W*_wet_) and then dried at 60°C in an oven for 3 days. The totally dried hydrogels were taken out and weighed (*W*_dry_) again. The water content (*W*_H2O_) was obtained from the following equation: *W*_H2O_ = (*W*_wet_ − *W*_dry_)/*W*_wet_.

### Measurement of weight swelling ratio

The as-prepared hydrogels were first weighed (*W*_0_) and then immersed into PBS at 37°C. After the predetermined time, the swollen hydrogels were taken out and weighed (*W*_s_) again. The swelling ratio (SR) was obtained from the following equation: SR = (*W*_s_ − *W*_0_)/*W*_0_.

## Supplementary Material

http://advances.sciencemag.org/cgi/content/full/5/2/eaau3442/DC1

## SUPPLEMENTARY MATERIALS

Supplementary material for this article is available at http://advances.sciencemag.org/cgi/content/full/5/2/eaau3442/DC1 Fig. S1. Microscopic network and tensile mechanical properties of conjoined-network hydrogels. Fig. S2. Biocompatibility of C4-G20-P20 conjoined-network hydrogel. Fig. S3. Mechanical properties of C4-G20 composite hydrogel. Fig. S4. Fracture energy of C-G-P conjoined-network hydrogels. Fig. S5. The dissipative capacity and fatigue resistance behavior of C4-G20 composite hydrogel. Fig. S6. Fatigue resistance and self-recovery behavior of C4-G20-P20 conjoined-network hydrogel under human body temperature conditions (37°C). Fig. S7. Precipitate formation by chitosan with various phosphates and effect of soaking media pH on mechanical behavior of conjoined-network hydrogel. Fig. S8. The effect of weight ratio of the first network to the second network on the mechanical properties and the swelling properties of C-G-P conjoined-network hydrogels. Fig. S9. Compressive stress-strain curve of the gelatin hydrogel without sodium phytate at a similar solid content to those C*x*-G*y*-P20 conjoined-network hydrogels and tunable mechanics (compressive modulus and toughness) of C-G-P conjoined-network hydrogels. Table S1. Quantitative comparison of the mechanical properties of C-G-P conjoined-network hydrogels with other natural polymer hydrogels, synthetic polymer hydrogels, and articular cartilage. Movie S1. This movie showing the stiff and tough C4-G20-P20 conjoined-network hydrogel can be used as a structural material to protect fragile objects (for example, an egg). Movie S2. This movie was shot at the same time with movie S1 at a close range.
