# Supplementary material for: Conjoined-network rendered stiff and tough hydrogels from biogenic molecules
Source: Sci Adv. 2019 Feb 1;5(2):eaau3442. doi: 10.1126/sciadv.aau3442 (PMC6358320; doi:10.1126/sciadv.aau3442)
Supplement: http://advances.sciencemag.org/cgi/content/full/5/2/eaau3442/DC1 [file supp_5_2_eaau3442__index.html]

Science Advances | Science Advances

## Supplementary Materials

**The PDF file includes:**

- Fig. S1. Microscopic network and tensile mechanical properties of conjoined-network hydrogels.
- Fig. S2. Biocompatibility of C4-G20-P20 conjoined-network hydrogel.
- Fig. S3. Mechanical properties of C4-G20 composite hydrogel.
- Fig. S4. Fracture energy of C-G-P conjoined-network hydrogels.
- Fig. S5. The dissipative capacity and fatigue resistance behavior of C4-G20 composite hydrogel.
- Fig. S6. Fatigue resistance and self-recovery behavior of C4-G20-P20 conjoined-network hydrogel under human body temperature conditions (37°C).
- Fig. S7. Precipitate formation by chitosan with various phosphates and effect of soaking media pH on mechanical behavior of conjoined-network hydrogel.
- Fig. S8. The effect of weight ratio of the first network to the second network on the mechanical properties and the swelling properties of C-G-P conjoined-network hydrogels.
- Fig. S9. Compressive stress-strain curve of the gelatin hydrogel without sodium phytate at a similar solid content to those C*x*-G*y*-P20 conjoined-network hydrogels and tunable mechanics (compressive modulus and toughness) of C-G-P conjoined-network hydrogels.
- Table S1. Quantitative comparison of the mechanical properties of C-G-P conjoined-network hydrogels with other natural polymer hydrogels, synthetic polymer hydrogels, and articular cartilage.

Download PDF

**Other Supplementary Material for this manuscript includes the following:**

- Movie S1 (.mp4 format). This movie showing the stiff and tough C4-G20-P20 conjoined-network hydrogel can be used as a structural material to protect fragile objects (for example, an egg).
- Movie S2 (.mp4 format). This movie was shot at the same time with movie S1 at a close range.

**Files in this Data Supplement:**

- Adobe PDF - aau3442\_SM.pdf
