# Supplementary material for: Conjoined-network rendered stiff and tough hydrogels from biogenic molecules
Source: Sci Adv. 2019 Feb 1;5(2):eaau3442. doi: 10.1126/sciadv.aau3442 (PMC6358320; doi:10.1126/sciadv.aau3442)
Supplement: http://advances.sciencemag.org/cgi/content/full/5/2/eaau3442/DC1 [file aau3442_SM.pdf]

Science Advances
AAAS

advances.sciencemag.org/cgi/content/full/5/2/eaau3442/DC1

Supplementary Materials for

Conjoined-network rendered stiff and tough hydrogels from biogenic molecules

Liju Xu, Chen Wang, Yang Cui, Ailing Li, Yan Qiao*, Dong Qiu*

*Corresponding author. Email: yanqiao@iccas.ac.cn (Y.Q.); dqiu@iccas.ac.cn (D.Q.)

Published 1 February 2019, *Sci. Adv.* **5**, eaau3442 (2019)
DOI: 10.1126/sciadv.aau3442

The PDF file includes:

Fig. S1. Microscopic network and tensile mechanical properties of conjoined-network hydrogels.
Fig. S2. Biocompatibility of C4-G20-P20 conjoined-network hydrogel.
Fig. S3. Mechanical properties of C4-G20 composite hydrogel.
Fig. S4. Fracture energy of C-G-P conjoined-network hydrogels.
Fig. S5. The dissipative capacity and fatigue resistance behavior of C4-G20 composite hydrogel.
Fig. S6. Fatigue resistance and self-recovery behavior of C4-G20-P20 conjoined-network hydrogel under human body temperature conditions (37°C).
Fig. S7. Precipitate formation by chitosan with various phosphates and effect of soaking media pH on mechanical behavior of conjoined-network hydrogel.
Fig. S8. The effect of weight ratio of the first network to the second network on the mechanical properties and the swelling properties of C-G-P conjoined-network hydrogels.
Fig. S9. Compressive stress-strain curve of the gelatin hydrogel without sodium phytate at a similar solid content to those C*x*-G*y*-P20 conjoined-network hydrogels and tunable mechanics (compressive modulus and toughness) of C-G-P conjoined-network hydrogels.
Table S1. Quantitative comparison of the mechanical properties of C-G-P conjoined-network hydrogels with other natural polymer hydrogels, synthetic polymer hydrogels, and articular cartilage.

Other Supplementary Material for this manuscript includes the following:

(available at advances.sciencemag.org/cgi/content/full/5/2/eaau3442/DC1)

Movie S1 (.mp4 format). This movie showing the stiff and tough C4-G20-P20 conjoined-network hydrogel can be used as a structural material to protect fragile objects (for example, an egg).
Movie S2 (.mp4 format). This movie was shot at the same time with movie S1 at a close range.

Supplementary Figures and Tables

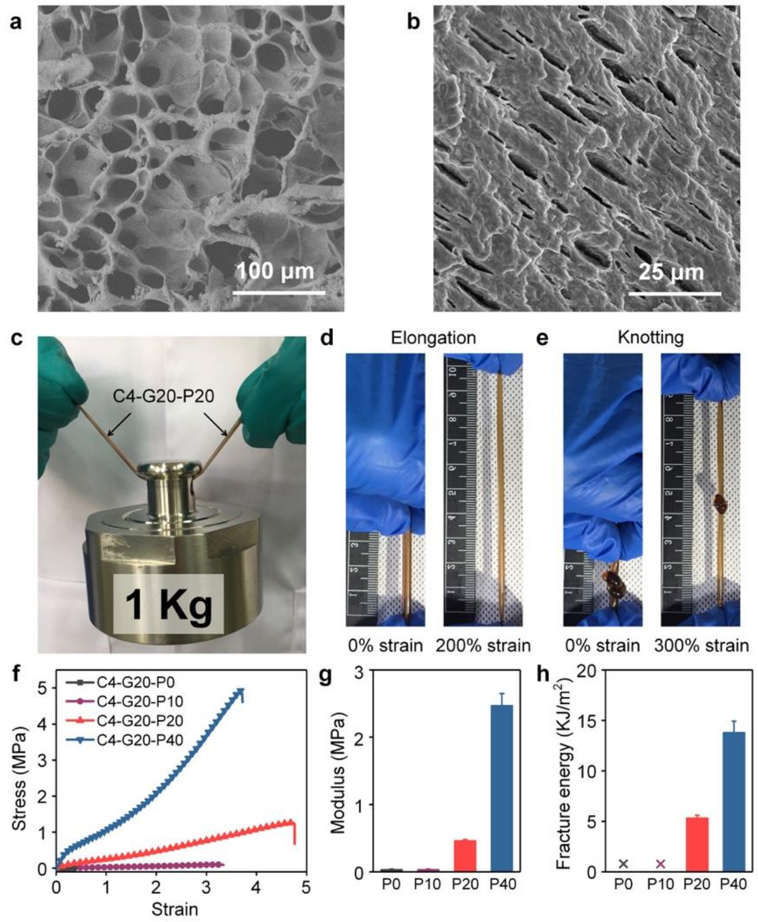

Fig. S1. Microscopic network and tensile mechanical properties of conjoined-network hydrogels. SEM images of (**a**) C4-G20-P0 and (**b**) C4-G20-P40 hydrogel. (**c**) The stiff and tough C4-G20-P20 hydrogel can sustain a large load of 1 kg. (**d**) Elongation, (**e**) knotting experiments of C4-G20-P20 hydrogel. (**f**) Tensile stress–strain curves, (**g**) modulus and (**h**) fracture energy of C4-G20-Pz hydrogels after soaking in sodium phytate solutions at various concentrations. The error bars represent standard deviation; sample size n = 3. The cross means not determined because the notched hydrogels were too fragile to measure the fracture energy. (Photo Credit: Liju Xu, Institute of Chemistry, Chinese Academy of Sciences).

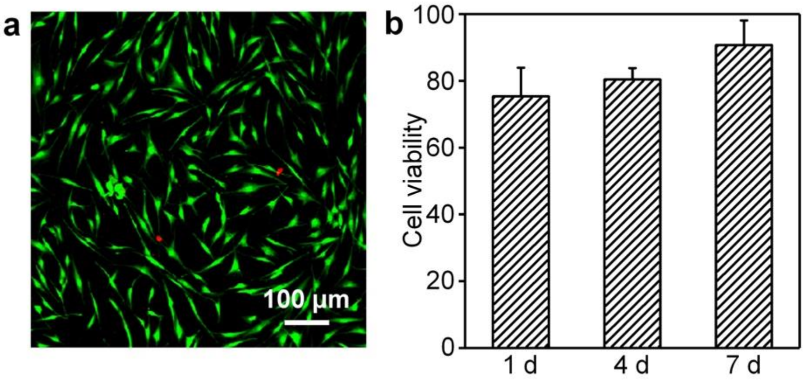

Fig. S2. Biocompatibility of C4-G20-P20 conjoined-network hydrogel. (**a**) Confocal fluorescence microscopy image of the human normal skin fibroblasts cells cultured on C4-G20-P20 hydrogel for 48 hours. (**b**) Cell viability of the human normal skin fibroblasts cells cultured on C4-G20-P20 hydrogel at day 1, 4 and 7 measured by CCK-8 method, showing cell viability of 75 ± 8%, 80 ± 3%, and 91 ± 7% after day 1, 4 and 7, respectively. The error bars represent standard deviation; sample size n = 5.

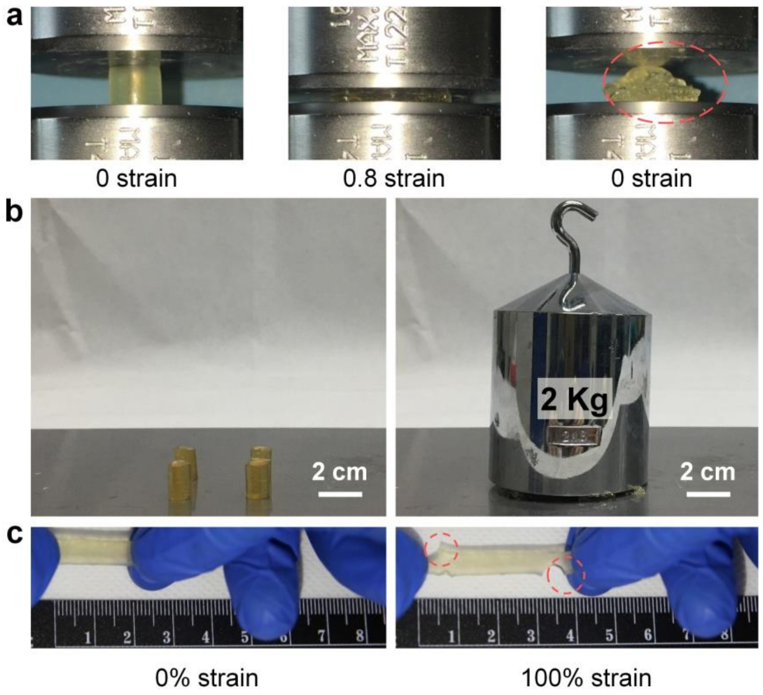

Fig. S3. Mechanical properties of C4-G20 composite hydrogel. (**a**) The C4-G20 composite hydrogel ruptured severely at 80% compressive strain. (**b**) The C4-G20 composite hydrogel was too soft to load a weight of 2 kg. (**c**) The C4-G20 composite hydrogel cannot be stretched over 100% strain, and some notches appeared. (Photo Credit: Liju Xu, Institute of Chemistry, Chinese Academy of Sciences).

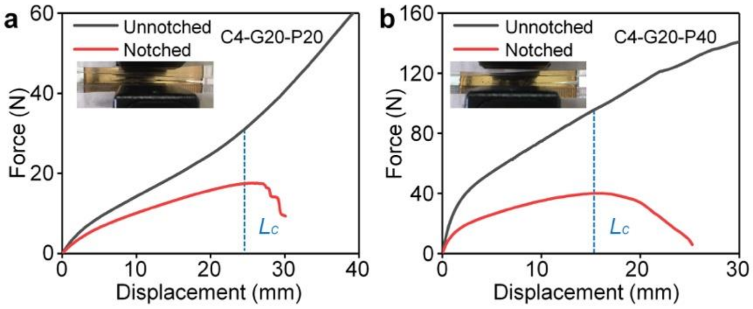

Fig. S4. Fracture energy of C-G-P conjoined-network hydrogels. The force-displacement curves of unnotched and notched (**a**) C4-G20-P20 and (**b**) C4-G20-P40 hydrogels. (Photo Credit: Liju Xu, Institute of Chemistry, Chinese Academy of Sciences).

Table S1. Quantitative comparison of the mechanical properties of C-G-P conjoined-network hydrogels with other natural polymer hydrogels, synthetic polymer hydrogels and articular cartilage. The C-G-P conjoined-network hydrogel possessed an outstanding combination of high compressive modulus and toughness. E, σ, Ɛ, U_T, W and Γ are the initial modulus, fracture stress, fracture strain, compressive toughness, work of extension and fracture energy under the compression and tension model, respectively.

**Hydrogel Type**		**Compression**				**Tension**				***Γ* (KJ/m^2)**	**Ref.**
		***E* (MPa)**	**σ (MPa)**	**Ɛ**	**U_T (MJ/m^3)**	***E* (MPa)**	**σ (MPa)**	**Ɛ**	**W (MJ/m^3)**		
Present work	Chitosan-Gelatin-Phytate	6.60	64.0	0.87	5.5	2.47	4.3	3.5	7.4	13.8	
Natural polymer based hydrogels	Gelatin-Am	0.45	13.0	0.99	~0.6 [a]	0.76	4.3	4.2	~4.8 [b]		34
	Chitin	0.22	4.0	0.81	0.4						10
	Cellulose	0.61	4.8	0.74	~0.5 [c]	2.00	2.7	0.8	0.8		11
	Silk	5.89	12.4	0.80	2.2						35
	DCC-alginate					367.40	19.8	0.5	7.0		36
Synthetic polymer based hydrogels	Alginate-polyacrylamide					0.06	0.2	17.0		9.0	24
	P(NaSS-co-MPTC)					2.05	1.8	7.0	7.1	4.0	25
	mineralized PAAm-l-MBAm					150.00	1.4	0.2		1.3	33
	PEDOT/PAMPS/PAAm	0.34	76.1	0.98	~2.4 [d]						26
	PVA-ANF	4.00	26.5	0.95	~3.0 [e]	9.10	5.0	3.3		9.2	37
	Articular cartilage	0.5-10	10-50			1-10	1-20			0.5-1.5	37

The value of compressive toughness and work of extension was estimated from the stress–strain curve in [a, b] ref. 34 Fig. 3a, b; [c] ref. 11 Fig. 5a; [d] ref. 26 Fig. 5a and [e] ref. 37 Fig. 3b.

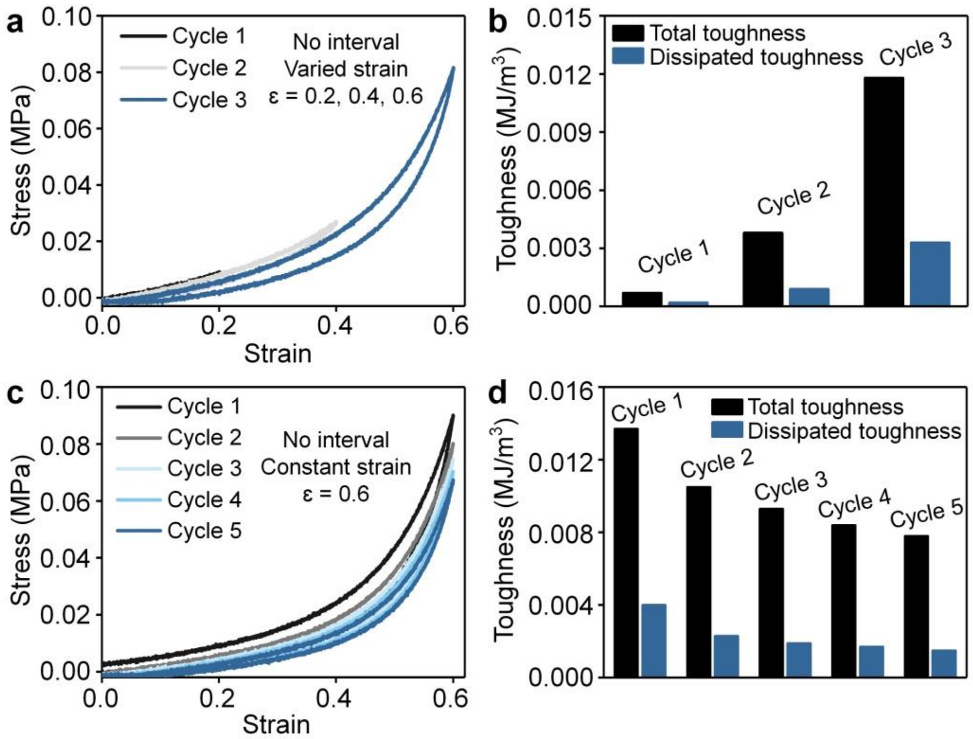

Fig. S5. The dissipative capacity and fatigue resistance behavior of C4-G20 composite hydrogel. (**a**) Sequential loading–unloading compression tests without interval and (**b**) the corresponding calculated total and dissipated toughness of the C4-G20 composite hydrogel under different strains (ε = 0.2, 0.4, 0.6). The C4-G20 composite hydrogel showed small hysteresis loops, indicating that the dissipative capacity of it was very weak. (**c**) Fatigue resistance and (**d**) the corresponding calculated total and dissipated toughness of the C4-G20 composite hydrogel with five successive loading-unloading cycles without interval under a constant strain (ε = 0.6). There was substantial reduction of compressive strength and dissipated energy for the C4-G20 composite hydrogel after five successive loading-unloading cyclic compressive tests.

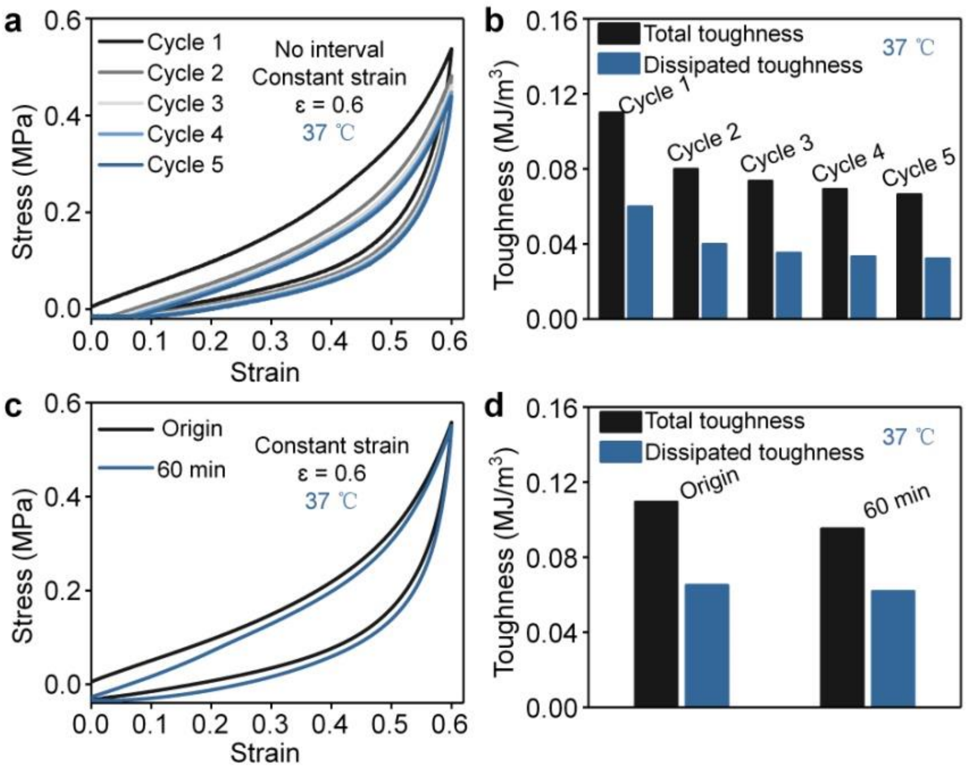

Fig. S6. Fatigue resistance and self-recovery behavior of C4-G20-P20 conjoined-network hydrogel under human body temperature conditions (37 °C). (**a**) Fatigue resistance and (**b**) the corresponding calculated total and dissipated toughness of the C4-G20-P20 hydrogel with five successive loading-unloading cycles without interval under a constant strain (ε = 0.6). (**c**) Recovery cyclic compression tests and (**d**) the corresponding calculated total and dissipated toughness of the C4-G20-P20 hydrogel for 60 min relaxation time under a constant strain (ε = 0.6).

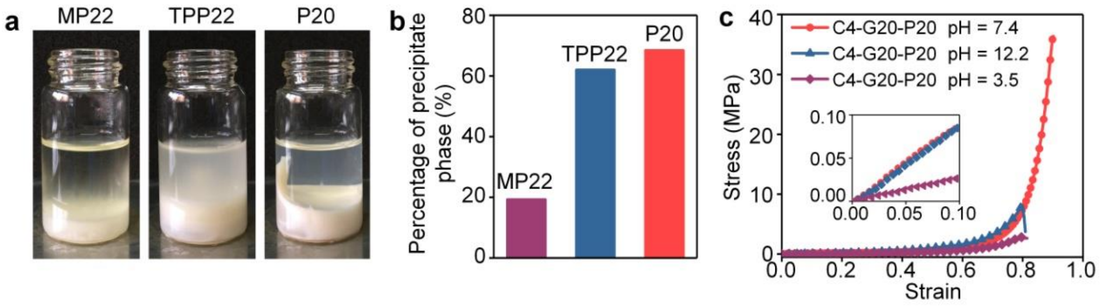

Fig. S7. Precipitate formation by chitosan with various phosphates and effect of soaking media pH on mechanical behavior of conjoined-network hydrogel. (**a**) Photographs of white precipitates formed by directly mixing chitosan solution (2 wt %) and various phosphates with different numbers of phosphate group (MA, TPP, P, mole concentrations of phosphate group were identical to 20 wt % sodium phytate, pH = 7.4). (**b**) The ratio of precipitate phase of chitosan to total chitosan in original solution. (**c**) Modulate conjoined-network of C4-G20-P20 hydrogel by adjusting soaking media pH. (Photo Credit: Liju Xu, Institute of Chemistry, Chinese Academy of Sciences).

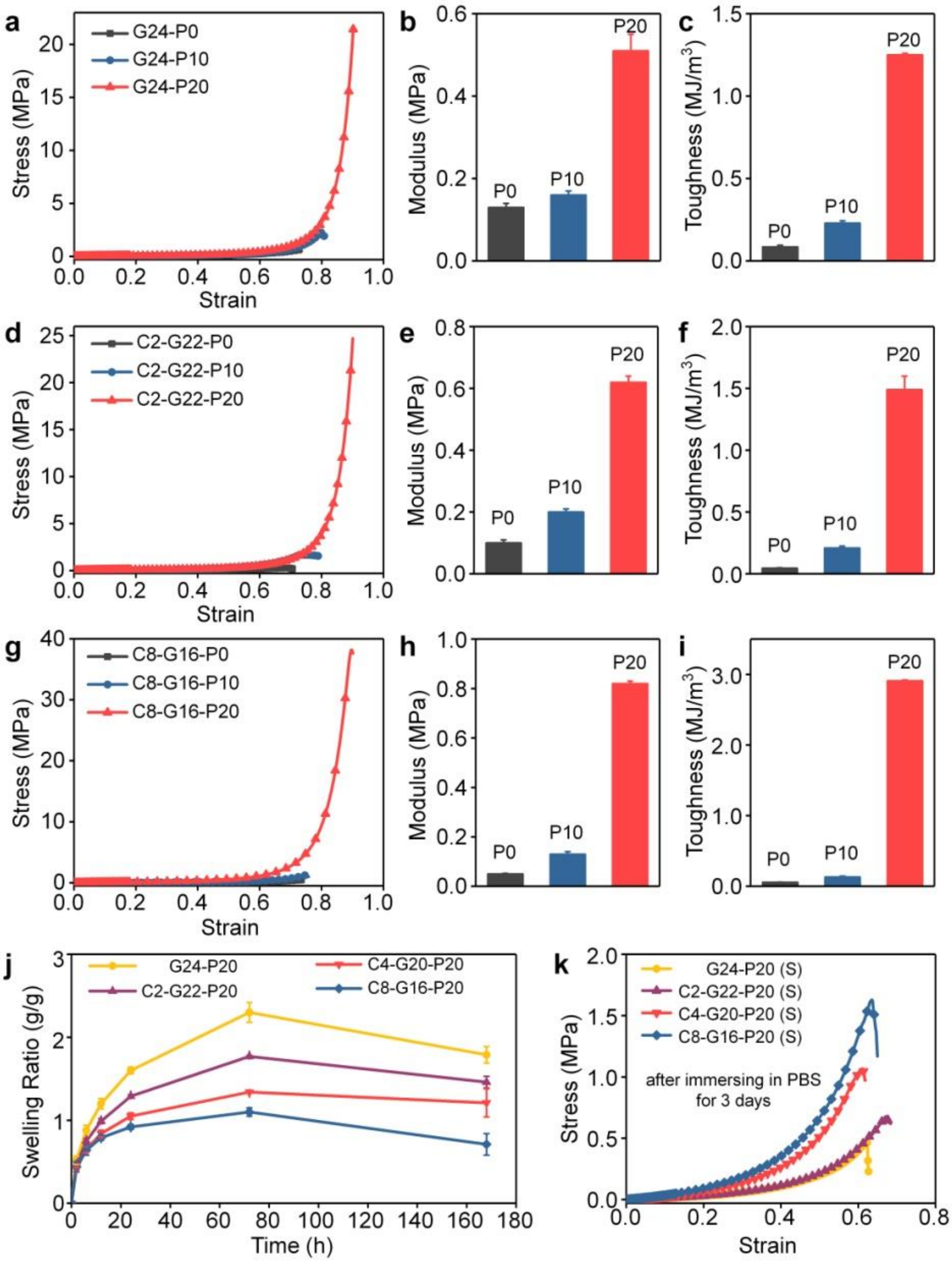

Fig. S8. The effect of weight ratio of the first network to the second network on the mechanical properties and the swelling properties of C-G-P conjoined-network hydrogels. (**a**, **d**, **g**) Compressive stress–strain curves, (**b**, **e**, **h**) initial modulus and (**c**, **f**, **i**) compressive toughness of Cx-Gy-Pz hydrogels with different weight ratio of the first network to the second network after soaking in sodium phytate solutions at various concentrations (pH = 7.4). (**j**) Swelling ratio of Cx-Gy-P20 hydrogels after immersing in PBS buffer at 37 ℃ for different time. (**k**) Compressive stress–strain curves of Cx-Gy-P20 hydrogels after immersing in PBS at 37 ℃ for 3 days. The error bars represent standard deviation; sample size n = 3.

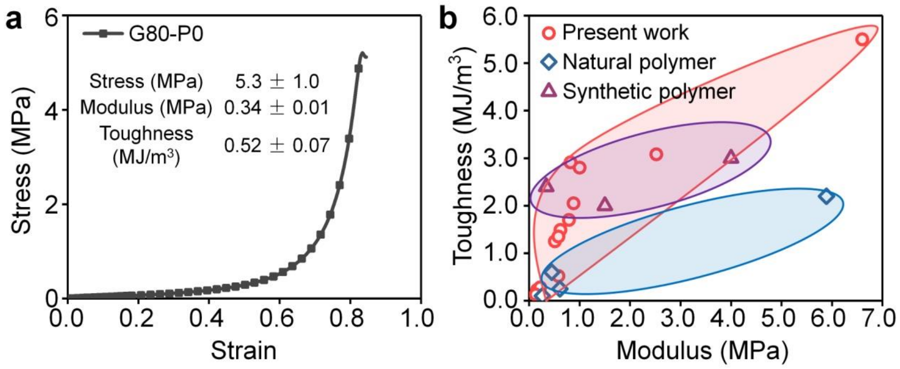

Fig. S9. Compressive stress-strain curve of the gelatin hydrogel without sodium phytate at a similar solid content to those C*x*-G*y*-P20 conjoined-network hydrogels and tunable mechanics (compressive modulus and toughness) of C-G-P conjoined-network hydrogels. (**a**) Compressive stress–strain curve of the gelatin hydrogel without sodium phytate at similar solid content to those Cx-Gy-P20 hydrogels. The error bars represent standard deviation; sample size n = 3. (**b**) Compressive modulus and toughness of C-G-P conjoined-network hydrogels. The mechanical properties of the C-G-P conjoined-network hydrogels can be tuned over a broad range, which readily achieved by simply adjusting concentration of soaking media, functionality of cross-linkers, the weight ratio of the first network to the second network or diverse ionic combination. The data of natural polymer based hydrogel and synthetic polymer based hydrogel in the figure came from Table S1.
